# Homœopathy and the Chicago City Hospital

**Published:** 1858-01

**Authors:** 


### HYMENIAL.

Married, in Orange, New Jersey, Nov. 28th 1857, by the Rev. F. A. Adams, D.D., J. B. Moffett, M.D., of Mineral Point, Wis., to Miss H. A. Larned, of Watertown, N. Y.

